# Long‐Term Survival and Prognostic Factors of Stereotactic Body Radiotherapy Following Transarterial Chemoembolization for Hepatocellular Carcinoma

**DOI:** 10.1002/cnr2.70212

**Published:** 2025-05-08

**Authors:** Hoang Dong Duc, Mai Binh Thanh, Mai Bang Hong, Nguyen Thinh Tien, Nguyen Thai Van, Bui Bieu Quang, Nguyen Chau Dinh, Thai Ky Doan

**Affiliations:** ^1^ Department of Internal Medicine Thai Nguyen University of Medicine and Pharmacy Thai Nguyen Vietnam; ^2^ Department of Gastroenterology and Hepatology 108 Military Central Hospital Hanoi Vietnam; ^3^ Department of Radiation Oncology and Radiosurgery 108 Military Central Hospital Hanoi Vietnam

**Keywords:** hepatocellular carcinoma, stereotactic body radiotherapy, transarterial chemoembolization

## Abstract

**Background/Aims:**

This study presents a detailed analysis of long‐term survival and critical factors influencing the outcomes of hepatocellular carcinoma patients treated with stereotactic body radiotherapy (SBRT) and transarterial chemoembolization (TACE). Our findings provide reassurance about the potential of the combination of TACE and SBRT as an effective treatment strategy for patients with large liver tumors due to HCC.

**Methods:**

A prospective study was conducted on 42 patients with intermediate‐stage hepatocellular carcinoma (HCC) at 108 Military Central Hospital between December 2018 and June 2024. Following a single session of TACE, each patient underwent SBRT 1 month later. The SBRT dose ranged from 27.5 to 48 Gy, delivered in 3–5 fractions. The patient survival analysis was conducted using the Kaplan–Meier method while examining prognostic factors influencing survival, which involved log‐rank tests and Cox proportional hazards regression analysis.

**Results:**

Among the 42 patients (83.3% male), 34 patients (81.0%) had tumors measuring ≥ 5 cm. The median follow‐up period was 32.2 months (4.5–65.1 months). The median overall survival (OS) was 32.6 months, with the respective 1‐, 3‐, and 5‐year OS rates reported as 73.8%, 24.5%, and 19.6%. Furthermore, the median progression‐free survival (PFS) was 16.6 months, with corresponding 1‐ and 3‐year PFS rates of 71.4% and 19.0%. Factors linked to improved OS and PFS included AFP levels and treatment response based on Modified RECIST criteria. Additionally, multivariate analysis identified patient age, EQD2, and BED10 as significant predictors of better survival outcomes.

**Conclusions:**

Our study provides evidence supporting the effectiveness and safety of combining TACE and SBRT as a treatment strategy for patients with large liver tumors due to HCC, instilling confidence in the future of HCC treatment. Positive prognostic factors included patient age, EQD_2_, and BED_10_.

**Abbreviations**
AFP: alpha‐fetoprotein
ASTRO: American Society for Radiation Oncology
BCLC: Barcelona Clinic liver cancer
BED: biologically effective dose
CR: complete response
CT: computed tomography
CTCAE: common terminology criteria for adverse events
DVH: Dose Volume Histogram
ECOG: Eastern Cooperative Oncology Group
EQ D_2_
: equivalent dose in 2 Gy fraction
Fr: fraction
GTV: gross tumor volume
HCC: hepatocellular carcinoma
ITV: internal target volume
MRECIST: modified response evaluation criteria in solid tumors
NCCN: National Comprehensive Cancer Network
OAR: organs at risk
OS: overall survival
*p*
: *p*‐value
PFS: progression‐free survival
PTV: planning target volume
RFA: radiofrequency ablation
RILD: radiation‐induced liver disease
SBRT: stereotactic body radiotherapy
TACE: transarterial chemoembolization

## 1 Introduction

Hepatocellular carcinoma (HCC) is ranked as the sixth most prevalent cancer globally, with 865 269 new cases and 757 948 reported deaths annually [1]. Early diagnosis of HCC is difficult due to its asymptomatic progression, limited public awareness, and the absence of routine screening, especially in high‐risk groups such as those with chronic liver inflammation and cirrhosis [2]. Even in advanced healthcare systems like those in the USA, Australia, Austria, and Korea, the rate of early detection remains below 50% [2, 3]. In Vietnam, the absence of a national screening program combined with the widespread prevalence of hepatitis B leads to the highest rates of both incidence and mortality. Early detection rates remain below 20% [4].

Tumor size significantly affects treatment outcomes, with larger tumors generally associated with a poorer prognosis. For liver tumors greater than 5 cm, the overall survival (OS) is approximately 38 months following surgical intervention [5] and 28 months with transarterial chemoembolization (TACE) [6]. While TACE remains the primary treatment for intermediate‐stage HCC when curative options are not feasible [7], its effectiveness diminishes with repeated sessions, and the risk of liver function deterioration increases [8]. Integrating TACE with additional therapeutic approaches may help address these limitations [9, 10, 11, 12, 13].

Stereotactic body radiotherapy (SBRT) is an advanced radiotherapy technique that has shown promise in treating HCC, particularly for patients ineligible for RFA [14, 15]. Historically, its application has been limited due to concerns about radiation‐induced liver disease (RILD) [13]. However, technological advancements, including respiratory motion management and image‐guided delivery, have improved the precision and safety of SBRT, making it a potential alternative or adjunct to TACE [16]. Initial studies evaluating the combination of SBRT and TACE have reported encouraging outcomes, suggesting improved local tumor control and survival benefits compared to TACE alone. According to Chiang et al., SBRT + TACE for patients with Barcelona Clinic liver cancer (BCLC) stage B–C HCC resulted in a median OS of 19.8 months [17], highlighting its potential as a viable treatment option.

Despite these promising results, evidence directly comparing SBRT + TACE to other combination strategies, such as TACE + RFA or TACE + systemic therapy, remains limited. While TACE + SBRT has demonstrated favorable outcomes in select patient populations, further research is needed to determine its comparative efficacy, long‐term survival benefits, and optimal patient selection criteria. Our study builds upon existing research by evaluating the long‐term survival outcomes and identifying prognostic factors for HCC patients who underwent SBRT following TACE. The findings, particularly identifying positive prognostic factors and high survival rates at 1, 3, and 5 years, suggest that combining TACE and SBRT could significantly improve the long‐term survival of HCC patients, especially those with significant liver tumors. By addressing this knowledge gap, our study provides critical insights into the role of SBRT + TACE in the evolving treatment landscape for HCC.

## 2 Materials and Methods

### 2.1 Patient Eligibility

A prospective study was conducted on 42 patients at the 108 Military Central Hospital in Hanoi, Vietnam, a leading medical institution in the region known for its expertise in hepatocellular carcinoma treatment, between December 2018 and June 2024. The patient population was diverse, representing the typical demographics of HCC patients in the region, with a majority of the patients being male and the mean age being 60.86 years.

The inclusion criteria for the study required adult patients (≥ 18 years old) with intermediate‐stage HCC, as defined by the BCLC classification. Participants must have an ECOG performance status of 0 or 1, compensated liver function (Child‐Pugh A or B with a score of 7 points), a healthy liver volume greater than 700 cm^3^, and liver tumors located at least 2 cm away from the stomach or intestines. All patients underwent TACE followed by SBRT.

Exclusion criteria included: (i) prior treatment with selective internal radiation therapy using Yttrium‐90; (ii) pregnant or lactating women; (iii) heart failure, renal failure, respiratory failure; (iv) prothrombin time < 50%, platelets < 50 G/L; (v) active gastrointestinal bleeding; (vi) disagreed to participate.

### 2.2 Ethics Statement

The study was approved by the Institutional Review Board of 108 Military Central Hospital for conduct at the institute under approval number: DTDL.CN‐10/19 and adhered to the principles of the 1975 Declaration of Helsinki. All participants were required to provide written informed consent following a comprehensive explanation of the treatment methods (TACE and SBRT) and the study procedures.

### 2.3 TACE Treatment

Preparation for the procedure includes cleaning and shaving pubic hair, fasting in the morning, and placing a peripheral IV line with 0.9% sodium chloride solution. The chemical embolization uses 75 mg of Doxorubicin mixed with DC‐Beads at 37.5 mg/mL. After injecting 10 mL of 2% Lidocaine for local anesthesia, the Seldinger technique is used to insert a percutaneous arterial catheter into the right femoral artery. A 5Fr Yashiro catheter, guided by a 0.035 guidewire, is inserted through the femoral artery catheter and advanced into the celiac artery for angiography. A 2.7Fr Progreat microcatheter with a coaxial Guidewire is then used to access the tumor‐feeding artery selectively, and the chemical solution is injected under fluoroscopy. Once the tumor‐feeding artery is occluded, the catheter is retracted to the original celiac artery, and the angiographic scan is repeated using the same contrast dose.

### 2.4 SBRT Treatment

To perform SBRT, 1–3 months post‐TACE, the patient undergoes preparation, including a consultation with the Digestive Oncology Subcommittee to confirm the HCC diagnosis and determine the indication for stereotactic radiotherapy. During the 4D CT simulation, the patient lies supine, fixed with a vac‐lok, and places their hands on a wing board. A first scan, without contrast, covers the area from 5 cm above the liver dome to the L2‐3 intervertebral space with a 2.5 mm slice thickness. A second scan, with contrast, focuses on the region 3 cm above and below the tumor, with a 2.5 mm slice thickness.

We transfer all 4D CT images to the treatment planning system (TPS) for radiation treatment planning. The mobile tumor's gross tumor volume (GTV) and internal target volume (ITV) should be delineated. The planning target volume (PTV) is then defined by adding a 3–5 mm margin to the ITV to account for any uncertainties in tumor movement and setup. Identify organs at risk (OARs) on the noncontrast CT_ave sequence according to ICRU‐83 guidelines, including body contour, bones, healthy liver, gallbladder, stomach, duodenum, intestines, esophagus, heart, lungs, kidneys, spinal cord, skin, and chest wall. Prescribe a dose of 27.5Gy–50Gy/3–5 fractions using Eclipse v13.6 software (Siemens Healthineers company, Erlangen, Germany), ensuring the treatment dose covers ≥ 95% of the PTV. The NCCN guidelines suggest 27.5–50 Gy in 3–5 fractions based on phase II trials demonstrating high local control (~80%–90%) in hepatocellular carcinoma (HCC) and liver metastases. ASTRO aligns closely with the 48–54 Gy/3–5 fractions range, making it a widely accepted SBRT regimen. Evaluate the radiation dose to treatment volume and healthy organs using the Dose Volume Histogram (DVH) diagram.

Radiation treatment plan verification: From the approved SBRT plan, use the tool (Create Verification Plan) in Eclipse v13.6 software to create a calculated dose plane matrix on EPID. Perform image‐guided radiotherapy using TrueBeam STx (Varian, USA) with the same posture and fixation as when taking a simulated CT scan. The conventional formula takes into account the biologically effective dose (BED), and the equivalent dose in 2‐Gy fractions (EQD_2_) is calculated assuming an α/β ratio of 10 for rapidly proliferating tumor cells and 3 for normal tissues. These ratios account for the differential response of tumors and normal tissues to radiation, helping to optimize treatment planning.

$$\mathrm{BED}=d\times n\left(1+\;d/\left(\alpha /\beta \right)\right)$$

$$\mathrm{EQD}=d\times n\left(\left(\alpha /\beta +d\right)/\left(\alpha /\beta +\mathrm{dx}\right)\right)$$

*d* = dose, *n* = fraction and *dx* = 2.

### 2.5 Follow Up and Evaluate Treatment Response

Following SBRT, patients were scheduled for follow‐up appointments at 1 month and subsequently every 3 months. Physical exams, lab tests, tumor markers, and triphasic CT scans were conducted. After the treatment, the serum levels of Alpha‐fetoprotein (AFP) posttreatment and those prior to the treatment were compared. AFP response was assessed in patients with elevated pretreatment AFP, considering a response if AFP normalized or decreased by at least 50% within 3 months after SBRT.

The assessment of tumor response was carried out at 3 and 6 months posttreatment, employing the mRECIST criteria [18]. An objective response, or treated response, was defined as achieving either a complete or partial response, while a complete response, partial response, or stable disease characterized disease control.

The assessed survival metrics included progression‐free survival (PFS), which was calculated from the time of SBRT to the occurrence of tumor progression, recurrence, metastasis, or venous thrombosis, and OS, which was measured from the time of SBRT to either death or the end of the study, with the study end point being 5 years after treatment. Survival rates were analyzed at follow‐up points using the Log Rank test. Radiation‐induced side effects were graded according to The National Cancer Institute Common Terminology Criteria for Adverse Events (CTCAE V5.0) for symptoms like abdominal pain, fever, fatigue, nausea, vomiting, and gastrointestinal bleeding.

OS and PFS were the primary endpoints for assessing long‐term survival of the treatment in this study. Secondary endpoints for assessing the effectiveness of the treatment were tumor response and adverse events.

### 2.6 Statistical Analysis

We presented the information using percentages and averages. We used the *χ* ^2^‐test for counting data. To calculate the probability of survival, we chose to use the Kaplan–Meier method. We utilized Log‐rank tests and a Cox regression model to execute prognostic univariate and multivariate analyses. The definition of OS encompassed the duration from the date of death or the conclusion of the follow‐up period for patients who underwent SBRT treatment. PFS was the time from SBRT treatment to disease progression or death. All statistical analyses were conducted using SPSS version 22 software (IBM Corp., Chicago, USA). A *p*‐value < 0.05 was significant in all analyses. The variables included in both the univariate and multivariate analyses were derived from published studies focusing on this treatment as well as studies related to other treatments for HCC patients.

## 3 Results

### 3.1 Patient Characteristics

In total, 42 HCC patients underwent TACE followed by SBRT, and their progress was monitored for a median period of 32.2 months (ranging from 4.5 to 65.1 months) after receiving SBRT. Their baseline characteristics, with a predominance of male patients (83.3%), are detailed in Table 1. Additionally, it was observed that 73.8% of the patients had hepatitis B infection, while almost all patients had an ECOG score of 0 (97.6%). A notable proportion of the patients, accounting for 52.4%, were newly diagnosed with HCC. Additionally, within the study population, there were 25 patients in BCLC stage B2, and most patients had solitary tumors (92.9%), with 81% having tumors larger than 5 cm. In most cases, approximately 57.1% of patients exhibited AFP levels below 200. HCC patients who received SBRT were primarily treated with five fractions, mostly with EQD2 < 74 Gy (83%) and BED10 < 100 Gy (83%).

**TABLE 1:** Baseline characteristics of patients.

	Number of patients or mean
Patient characteristics	
< 60 years	17 (40.5)
≥ 60 years	25 (59.5)
Sex, *n* (%)	
Male	35 (83.3)
Female	7 (16.7)
Etiology, *n* (%)	
Hepatitis B	31 (73.8)
Hepatitis C	2 (4.8)
Hepatitis B,C	1 (2.4)
Unknown	8 (19.0)
ECOG, *n* (%)	
0	41 (97.6)
1	1 (2.4)
Treatment, *n* (%)	
Primary	22 (52.4)
Recurrent	20 (47.6)
Child‐Pugh class, *n* (%)	
A	41 (97.6)
B	1 (2.4)
BCLC‐B subclassification, *n* (%)	
B1	17 (40.5)
B2	25 (59.5)
Number of tumors, *n* (%)	
Solitary	39 (92.9)
Multiple	3 (7.1)
Tumor size, *n* (%)	
< 5 cm	8 (19.0)
≥ 5 cm	34 (81.0)
AFP, *n* (%)	
< 200 ng/mL	24 (57.1)
≥ 200 ng/mL	18 (42.9)
Total dose/fraction, *n* (%)	
30–48 Gy/3 Fr	10 (23.8)
27.5–42.5 Gy/5 Fr	32 (76.2)
EQD_2_	
< 74 Gy	35 (83.3)
≥ 74 Gy	7 (16.7)
BED_10_	
< 100 Gy	35 (83.3)
≥ 100 Gy	7 (16.7)

Abbreviations: AFP, alpha‐fetoprotein; BCLC, Barcelona Clinic liver cancer; BED, biologically effective dose; ECOG, Eastern cooperative oncology group; EQD_2_, equivalent dose in 2Gy fraction; HBV, hepatitis B virus; HCV, hepatitis C virus.

### 3.2 Treatment Response

Tumor response was evaluated in 42 patients at 3 and 6 months on contrast‐enhanced CT images. The results showed CR (50.0%), PR (7.5%) and SD (10.0%), and PD (32.5%), and the mean tumor size decreased from 7.06 ± 2.20 cm before treatment to 4.85 ± 1.98 cm at 6 months after treatment (Table 2).

**TABLE 2:** Tumor response after treatment.

Time point	Tumor size (cm) mean ± SD	*p*	Number patient	mRECIST, *n* (%)			
				CR	PR	SD	PD
Baseline (1)	7.06 ± 2.20		42	—	—	—	—
1 month (2)	5.41 ± 1.97	*p* ^2−1^ = 0.001	42	12 (28.6)	4 (9.5)	21 (50.0)	5 (11.9)
3 months (3)	5.19 ± 2.06	*p* ^3−1^ = 0.001	42	17 (40.5)	3 (7.1)	12 (28.6)	10 (23.8)
6 months (4)	4.85 ± 1.98	*p* ^4−1^ = 0.001	40	20 (50.0)	3 (7.5)	4 (10.0)	13 (32.5)

Abbreviations: CR, complete response; mRECIST, response evaluation criteria in solid tumors; PD, progressive disease; PR, partial response; SD, stable disease.

### 3.3 Toxicity

The frequency of adverse events following treatment included fatigue, abdominal pain, nausea, acute hepatitis, pleural effusion, fever, and dermatitis, with most patients experiencing these symptoms at grade 1. Of the 42 patients, one reported grade 2 fatigue, one grade 2 fever, and one grade 2 abdominal pain. We observed no grade 3 acute toxicities (Table 3).

**TABLE 3:** Toxicity after treatment.

Toxicity	Grade		
	1	2	3
Abdominal pain	8 (19.0%)	1 (2.3%)	0
Fever	2 (4.7%)	1 (2.3%)	0
Fatigue	9 (21.4%)	1 (2.3%)	0
Vomiting	6 (14.2%)	0	0
Acute hepatitis	2 (4.7%)	0	0
Dermatitis	2 (4.7%)	0	0
Pleural effusion	3 (7.1%)	0	0

### 3.4 Prognostic Factors for OS and PFS

**FIGURE 1:**
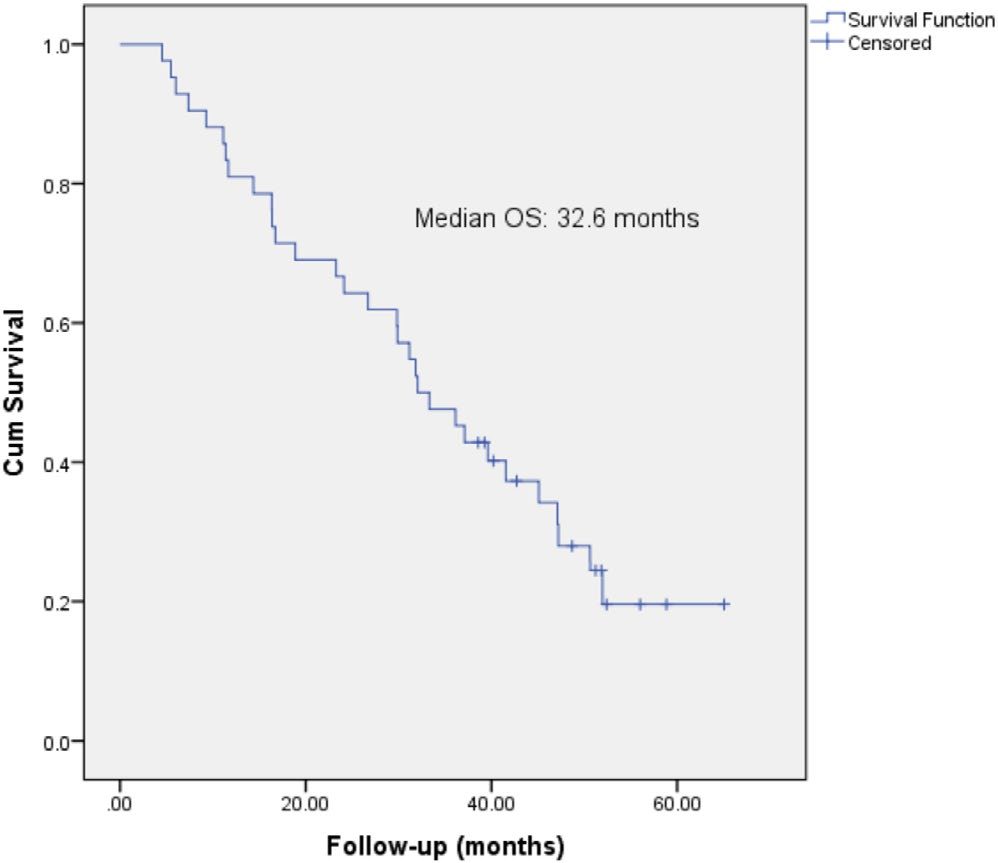
Kaplan–Meier survival curves; median overall survival was 32.6 months.

Following the combined treatment, the median survival was 32.6 months, with the 1‐, 3‐, and 5‐year OS rates at 73.8%, 24.5%, and 19.6%, respectively (Figure 1). The number of patients who died at 5 years of follow‐up was 31/42. The median PFS reached an impressive 16.6 months, with 1‐ and 3‐year PFS rates of 71.4% and 19.0%, respectively. The number of patients who were alive without disease progression was 8/42.

**FIGURE 2:**
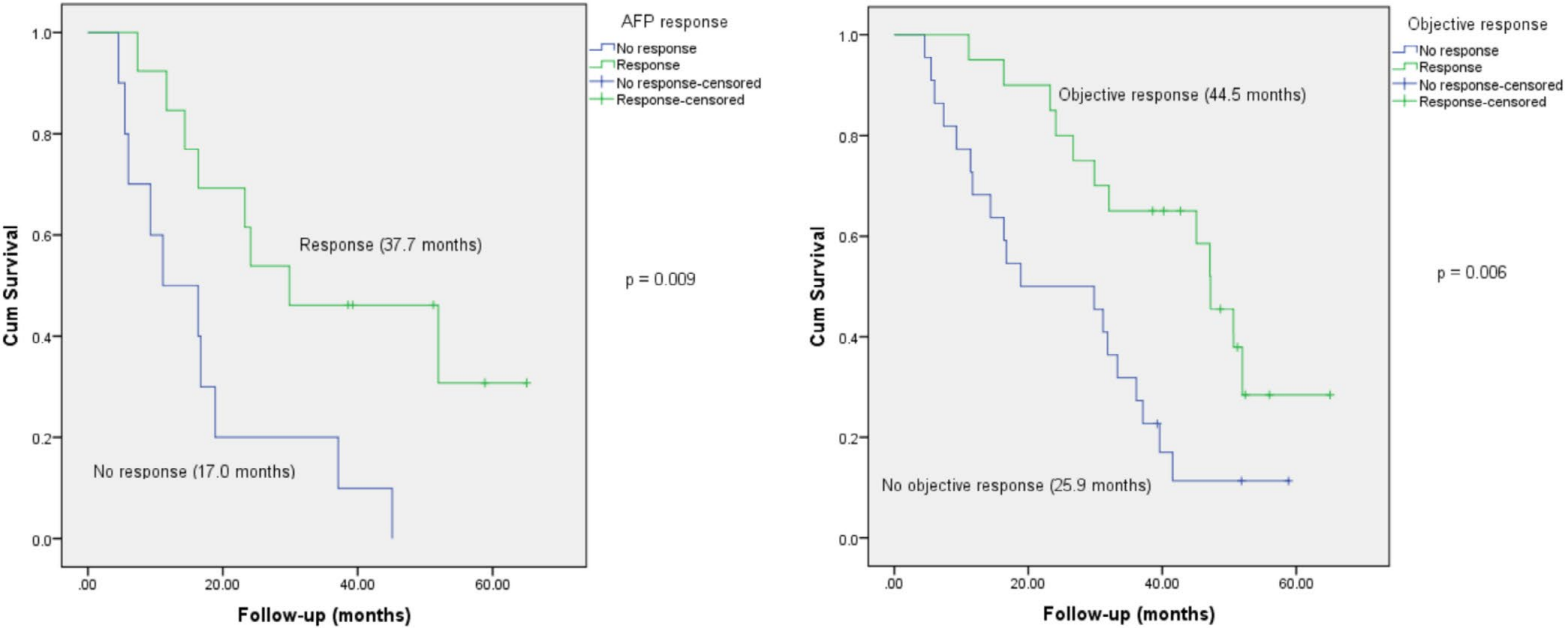
AFP response and objective response were significant prognostic factors for OS.

**FIGURE 3:**
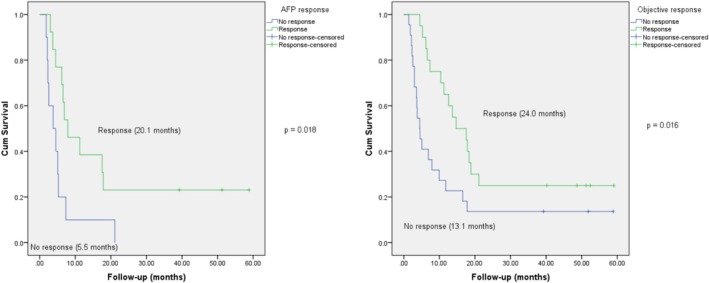
AFP response and objective response were significant prognostic factors for PFS.

Overall, the group with AFP response exhibited a higher OS time (median 37.7 months) compared to the unresponsive group (17.0 months), *p* = 0.009. Furthermore, patients who responded to treatment experienced prolonged survival (median 44.5 months) compared to nonresponders (25.9 months), *p* = 0.006 (Figure 2). Similarly, patients who showed decreased AFP and an objective response had significantly longer PFS compared to nonresponders (Figure 3).

Upon univariate analysis, a significant positive correlation was found between OS, PFS, and the AFP response, as well as the objective response. Meanwhile, other factors such as age, previous treatment, AFP levels, tumor size, disease stage, EQD_2_, and BED_10_ did not show a relationship with OS and PFS (Tables 4 and 5). On the contrary, the multivariate analysis indicated that age, EQD_2_, BED_10_, AFP response, and objective response were all notably independent predictors of both OS and PFS (Tables 4 and 5).

**TABLE 4:** Univariate analysis and multivariate analysis of variables predictive for progression‐free survival.

Variables		Univariate analysis HR (95% CI)	*p*	Multivariate analysis HR (95% CI)	*p*
Age	< 60 ≥ 60	1.63 (0.80–3.30) 1	0.17	0.08 (0.01–0.67)	0.01
Previous treatment	No Yes	0.86 (0.43–1.69) 1	0.66		
Alpha‐fetoprotein	< 200 ≥ 200	1.20 (0.60–2.37) 1	0.59		
Number of tumors	Solitary Multiple	1.78 (0.87–3.61) 1	0.11		
Tumor diameter (cm)	< 5 ≥ 5	0.61 (0.26–1.41) 1	0.25		
BCLC	B1 B2	0.90 (0.46–1.76) 1	0.77		
EQD_2_	< 74 Gy ≥ 74 Gy	1.84 (0.77–4.39) 1	0.16	0.92 (0.01–0.90)	0.04
BED_10_ (Gy)	< 100 Gy ≥ 100 Gy	1.84 (0.77–4.39) 1	0.16	0.92 (0.01–0.90)	0.04
AFP response	No Yes	0.35 (0.14–0.86) 1	0.02	2.77 (0.61–12.54)	0.18
Objective response	No Yes	0.44 (0.22–0.87) 1	0.01	2.78 (0.91–8.43)	0.70

Abbreviations: AFP, alpha‐fetoprotein; BCLC, Barcelona Clinic liver cancer staging system; CI, confidential index; EQD2, equivalent dose in 2 Gy fraction; HR, hazard ratio.

**TABLE 5:** Univariate analysis and multivariate analysis of variables predictive for overall survival.

Variables		Univariate analysis HR (95% CI)	*p*	Multivariate analysis HR (95% CI)	*p*
Age	< 60 ≥ 60	0.53 (0.24–1.15) 1	0.11	0.04 (0.01–0.33)	0.01
Previous treatment	No Yes	1.49 (0.72–3.08) 1	0.27		
Alpha‐fetoprotein	< 200 ≥ 200	0.70 (0.34–1.43) 1	0.33	0.09 (0.01–0.58)	0.01
Number of tumors	Solitary Multiple	0.61 (0.29–1.27) 1	0.19		
Tumor diameter (cm)	< 5 ≥ 5	0.92 (0.37–2.27) 1	0.87		
BCLC	B1 B2	1.05 (0.50–2.21) 1	0.88		
EQD_2_	< 74 Gy ≥ 74 Gy	1.29 (0.49–3.38) 1	0.60	0.05 (0.01–0.76)	0.03
BED_10_ (Gy)	< 100 Gy ≥ 100 Gy	1.29 (0.49–3.38) 1	0.60	0.05 (0.01–0.76)	0.03
AFP response	No Yes	3.44 (1.29–9.18) 1	0.01	7.74 (1.59–37.74)	0.01
Objective response	No Yes	2.75 (1.31–5.79) 1	0.01	9.32 (1.50–58.02)	0.01

Abbreviations: AFP, alpha‐fetoprotein; BCLC, Barcelona Clinic Liver Cancer staging system; CI, confidential Index; EQD2, equivalent dose in 2 Gy fraction; HR, hazard ratio.

## 4 Discussion

TACE is recommended for the treatment of intermediate‐stage HCC in patients whose tumors are not eligible for surgical removal or percutaneous ablation [7]. Additionally, TACE is also used as a downstaging or bridging therapy for liver transplant candidates, helping to manage the disease while they await transplantation [19]. As a result, TACE is more widely utilized in Asian countries, where different staging systems support its broader indication for HCC treatment [20]. However, for larger tumors (> 5 cm), multiple TACE sessions may be required, which can diminish the treatment's effectiveness and elevate the risk of liver function deterioration with each subsequent session [8]. Therefore, the current optimal strategy being considered is the combination of treatment methods, integrating TACE with additional treatments such as RFA, targeted therapy, or radiotherapy [9, 11, 12, 13, 17, 21, 22].

SBRT is gaining popularity because its precise radiation delivery offers optimal treatment efficacy while minimizing side effects. As a result, it is being applied to various types of cancer [13]. Combining SBRT after TACE has emerged as a relatively new treatment approach over the past decade. This combined therapy has shown promising results in achieving reasonable tumor control in unresectable HCC patients, underscoring the value of integrating these two treatment modalities [15, 16, 23]. We conducted a 5‐year prospective study with a median 32.2‐month follow‐up period on 42 patients with intermediate‐stage liver cancer in Vietnam to assess the effectiveness and safety of combining TACE and SBRT.

After TACE, patients received SBRT with doses ranging from 27.5 to 48 Gy, split into 3–5 fractions. These results are similar to reports from other authors worldwide, such as Lee and Jiang, who performed SBRT with doses of 30–50 Gy divided into 3–4 fractions [24, 25]. In addition, our patients mainly reported mild side effects following treatment, including grade 1 nausea, fatigue, and liver dysfunction. Only one patient experienced grade 2 fatigue, one grade 2 fever; another reported grade 2 abdominal pain. No grade 3 side effects were observed. Our result further supports the safety of combining TACE with SBRT, consistent with previously published reports [23, 24, 25].

In our assessment of the OS rate among 42 HCC patients, the mean OS was 32.6 months. The remarkable OS rates at 1 year, 3 years, and 5 years stood at 73.8%, 24.5%, and 19.6%, respectively. The study conducted by Su et al. found that the OS rates for the TAE/TACE + SBRT group were 75.5% at 1 year, 50.8% at 3 years, and 46.9% at 5 years. However, it is important to note that the median follow‐up time in that study was 20.5 months, which is shorter than the follow‐up period in this study [26]. Comparatively, a study involving 57 patients treated with SBRT following incomplete TACE reported a median OS of 26.6 months, with 1‐year, 2‐year, and 3‐year survival rates of 73.2%, 51.4%, and 32.4%, respectively [24]. Therefore, our treatment results after a 5‐year follow‐up are consistent with published reports [25, 27, 28] despite our study population mainly having large liver tumors (81% of our patients had liver tumors > 5 cm). Survival data were compared with previous SBRT + TACE studies (Table 6). This indicates that combining SBRT to target residual liver tumors after TACE is an effective option for treating large liver tumors, with ongoing improvements in the technical process enhancing its effectiveness.

**TABLE 6:** Comparison of survival data with SBRT combined with TACE.

Study	Treatment groups	Overall survival	Progression‐free survival	Key findings
Wong et al. [14]	TACE + SBRT (*n* = 49) vs. TACE alone (*n* = 98)	1‐year OS: 67.2% vs. 43.9% 3‐year OS: 36.5% vs. 13.3% *p* = 0.003	1‐year PFS: 32.5% vs. 21.4% 3‐year PFS: 15.1% vs. 5.1% *p* = 0.012	Combination of SBRT and TACE significantly improved OS and PFS in nonresectable HCC patients
Zhao et al. [15]	TACE + SBRT vs. SBRT alone	5‐year OS: 95% CI 1.01–2.04, *p* = 0.04	Not significantly different between the two groups	Combination of SBRT and TACE shows greater effectiveness than SBRT alone for treating unresectable HCC
Chiang et al. [17]	TACE + SBRT	The median OS was 19.8 months (95% CI, 11.6–30.6 months)	Not specified	TACE and SBRT can serve as safe and effective initial therapies for BCLC stage B‐C HCC with proper patient selection
Yao et al. [22]	TACE + SBRT	OS at 1, 2‐year: 75.8%, 45.5%	Not specified	SBRT is an effective noninvasive and palliative treatment option for patients with recurrent or residual HCC following TACE
Su et al. [26]	TACE + SBRT (*n* = 77) vs. SBRT alone (*n* = 50)	1‐year OS: 75.5% vs. 62.4% 3‐year OS: 50.8% vs. 32.9% 5‐year OS: 46.9% vs. 32.9% *p* = 0.047	Not significantly different between the two groups	Planned combination therapy resulted in higher CR rates and improved LC.

Variations in survival benefits among studies underline the need to identify prognostic factors for predicting the survival of patients undergoing TACE‐SBRT treatment. The variables included in the univariate and multivariate analyses were obtained from published studies of this treatment and studies of other treatments for HCC patients [24, 29]. Our study found that patients with AFP and favorable treatment responses demonstrated improved survival outcomes in terms of OS and PFS. These findings are consistent with those of similar studies conducted worldwide. Author Jiang and colleagues reported that patients with more than 75% AFP reduction had longer OS than patients with less than 75% AFP reduction after SBRT (*p* = 0.018) [24]. In addition, our thorough analysis revealed that being under 60 years old, having an EQD2 of less than 74 Gy, and a BED10 of less than 100 Gy were identified as critical factors independently contributing to better OS and PFS in our patient group. Our study's independent prognostic factors for OS and PFS may differ from previous studies. Wong et al. reported that an AFP level greater than 200 ng/mL, large tumors, and multiple tumors were predictive of worse OS [14]. This could be due to differences in the population or patient baseline characteristics. However, these independent prognostic factors are valuable for predicting treatment outcomes in liver cancer patients undergoing combined TACE and SBRT therapy.

Our study has several limitations. Firstly, the small sample size is attributable to the novelty of SBRT as a treatment technique for HCC in Vietnam, which relies on the TrueBeam radiotherapy system. It has not been included in the national treatment guidelines and is not covered by health insurance, limiting patient treatment options. Secondly, it is worth noting that this study was conducted at the 108 Central Military Hospital, which could potentially limit the generalizability of the findings. To better assess the efficacy and prognostic factors of TACE and SBRT treatment outcomes, we recommend conducting this study across multiple centers with a larger patient population.

## 5 Conclusion

The combination of TACE and SBRT is a potentially effective and safe treatment approach for patients with intermediate‐stage HCC presenting with large tumors. Age, EQD_2_, and BED_10_ are independent prognostic factors influencing the treatment effectiveness of this combined therapy.

## Author Contributions

**Hoang Dong Duc:** conceptualization (equal), formal analysis (equal), methodology (equal), visualization (equal), writing – original draft (equal). **Mai Binh Thanh:** conceptualization (equal), formal analysis (equal), methodology (equal), visualization (equal), writing – review and editing (equal). **Mai Bang Hong:** conceptualization (equal), formal analysis (equal), methodology (equal). **Nguyen Thinh Tien:** conceptualization (equal), formal analysis (equal), methodology (equal). **Nguyen Thai Van:** conceptualization (equal), formal analysis (equal), methodology (equal). **Bui Bieu Quang:** conceptualization (equal), formal analysis (equal), methodology (equal). **Nguyen Chau Dinh:** conceptualization (equal), formal analysis (equal), methodology (equal). **Thai Ky Doan:** conceptualization (equal), formal analysis (equal), methodology (equal), visualization (equal), writing – review and editing (equal).

## Ethics Statement

We hereby confirm that the aforementioned manuscript has not been previously published.

## Conflicts of Interest

The authors declare no conflicts of interest.

## Data Availability

Due to the privacy policy of 108 Military Central Hospital, the datasets collected and analyzed in the course of this study are not intended for public dissemination. However, we are prepared to furnish the data upon receipt of a substantiated request.
